# Human genetics of mycobacterial disease

**DOI:** 10.1007/s00335-018-9765-4

**Published:** 2018-08-16

**Authors:** Monica Dallmann-Sauer, Wilian Correa-Macedo, Erwin Schurr

**Affiliations:** 1 Program in Infectious Diseases and Immunity in Global Health, Research Institute, McGill University Health Centre, Montreal, QC Canada; 2 The McGill International TB Centre, McGill University, Montreal, QC Canada; 3 Departments of Human Genetics and Medicine, Faculty of Medicine, McGill University, Montreal, QC Canada; 4 Department of Biochemistry, Faculty of Medicine, McGill University, Montreal, QC Canada

## Abstract

Mycobacterial diseases are caused by members of the genus *Mycobacterium*, acid-fast bacteria characterized by the presence of mycolic acids within their cell walls. Claiming almost 2 million lives every year, tuberculosis (TB) is the most common mycobacterial disease and is caused by infection with *M. tuberculosis* and, in rare cases, by *M. bovis* or *M. africanum*. The second and third most common mycobacterial diseases are leprosy and buruli ulcer (BU), respectively. Both diseases affect the skin and can lead to permanent sequelae and deformities. Leprosy is caused by the uncultivable *M. leprae* while the etiological agent of BU is the environmental bacterium *M. ulcerans*. After exposure to these mycobacterial species, a majority of individuals will not progress to clinical disease and, among those who do, inter-individual variability in disease manifestation and outcome can be observed. Susceptibility to mycobacterial diseases carries a human genetic component and intense efforts have been applied over the past decades to decipher the exact nature of the genetic factors controlling disease susceptibility. While for BU this search was mostly conducted on the basis of candidate genes association studies, genome-wide approaches have been widely applied for TB and leprosy. In this review, we summarize some of the findings achieved by genome-wide linkage, association and transcriptome analyses in TB disease and leprosy and the recent genetic findings for BU susceptibility.

## Introduction

A common observation in human infectious diseases is that contact with pathogens is necessary but not sufficient for infection and subsequent development of clinical disease. Environmental and host factors, including the host genetic background, play a crucial role in the outcome of microbial exposure (Alcaïs et al. 2009; Casanova and Abel 2013). In mycobacterial diseases, an example that demonstrates the inherent spectrum of susceptibility to clinical disease is a tragic event known as the Lübeck disaster. In 1929, before the advent of antibiotics, 251 newborns were inoculated with Bacillus Calmette–Guérin (BCG) vaccine accidentally contaminated with virulent *Mycobacterium tuberculosis*—the etiological agent of tuberculosis (TB) (Fox et al. 2016). As a consequence, 228 infants developed clinical disease and 72 died from TB within a year of inoculation. Overall, 68% of those who had developed clinical TB recovered spontaneously, indicating natural resistance to TB. Also, analysis of the available data indicated that different vaccine batches were contaminated with different amounts of *M. tuberculosis*. It was observed that the bacterial dose had an important impact on the outcome of such exposure, since increased mortality could be attributed to increased levels of *M. tuberculosis* contamination. Of note, children who had been inoculated with similar amounts of *M. tuberculosis* displayed a broad spectrum of clinical symptoms ranging from total absence of clinical disease to death. This suggested that host-related factors, such as the genetic background, play an important role in innate resistance to TB (Fox et al. 2016). More direct evidence that human genetics can determine the outcome of mycobacterial infections comes from studies on Mendelian susceptibility to mycobacterial disease (MSMD), a rare condition characterized by selective predisposition to clinical disease caused by weakly virulent mycobacteria species. Studies of MSMD resulted in the identification of multiple rare single-gene mutations in genes encoding proteins of the IL12 and IFN-γ pathway as cause of susceptibility to mycobacterial disease (Boisson-Dupuis et al. 2015; Casanova 2015). At the population level, genetic studies have been performed to better understand the role of the human genetic background for the eventual outcome of mycobacterial infection, mainly TB and leprosy—the two most common mycobacterial diseases. Here, we provide an overview of selected findings obtained by genome-wide approaches used in the search of human genetic predisposition to clinical TB disease and leprosy. We also summarize initial genetic findings obtained for buruli ulcer (BU), the third most common mycobacterial disease.

## Tuberculosis

Tuberculosis is caused by *M. tuberculosis* and can affect any part of the body but most commonly affects the lungs leading to pulmonary TB (PTB). In 2016, the estimated TB incidence was 10.4 million new cases worldwide and nearly 1.7 million deaths due to the disease, including almost 0.4 million deaths among HIV-positive individuals (WHO 2017a). Exposure to *M. tuberculosis* leads to elimination of the pathogen and no persistent infection in 20–50% of individuals (Abel et al. 2014). Absence of persistent infection is inferred from a negative tuberculin skin test (TST) and/or IFN-γ release assay (IGRA) in individuals exposed to the pathogen (Pai et al. 2016). The identification of a major human genetic component linked to lack of TST reactivity in subjects exposed to *M. tuberculosis* expands the role of genetic factors to infection resistance (Cobat et al. 2009, 2015). Genetic findings of infection resistance to *M. tuberculosis* were recently reviewed elsewhere (Abel et al. 2017; Orlova and Schurr 2017; Simmons et al. 2018). Among individuals infected with *M. tuberculosis*, a person can remain in an asymptomatic state known as latent TB infection (LTBI) and never develop clinical disease or can evolve to clinical TB (active TB), whether it is primary TB or PTB (Abel et al. 2014; Pai et al. 2016). Primary TB is characterized by clinical disease that occur shortly after infection, either without LTBI or after a very short LTBI phase. While the majority of TB cases occur shortly (< 1 years) after infection (Sloot et al. 2014), among children progression of infection often leads to extrapulmonary, disseminated disease (severe primary TB). On the other hand, PTB occurs mostly in adults and is characterized by chronic pulmonary infection that can cause extensive lung damage (Abel et al. 2014; Pai et al. 2016). Different types of genetic control might explain these distinct outcomes of TB infection where, at one end of the spectrum, single-gene variations predispose individuals to severe childhood TB while older age disease involves more complex and polygenic factors (Alcaïs et al. 2005, 2010).

### Genome-wide linkage studies

Several genome-wide linkage studies (GWLS) identified chromosomal regions co-segregating with clinical TB within families (Table 1), but only in some of those regions have candidate genes been associated with disease in follow-up studies (Orlova and Schurr 2010). In families from South Africa and The Gambia, a two-stage GWLS revealed two suggestive linkage peaks on chromosomes 15q and Xq (Table 1) (Bellamy et al. 2000). An association study conducted in the same families from the GWLS and additional families from Guinea identified *UBE3A* on chromosome region 15q as a candidate gene for TB susceptibility (Cervino et al. 2002). In Brazilian families with PTB cases, three chromosomal regions—10q26.13, 11q12.3 and 20p12.1—showed suggestive evidence for linkage with disease (Table 1) (Miller et al. 2004). A GWL scan for PTB conducted in 48 Moroccan families followed by fine mapping linkage analysis of suggestive findings in an extended population of 96 families found chromosome region 8q12–q13 significantly linked to PTB (Table 1) (Baghdadi et al. 2006). In a follow-up study, 3216 SNPs within this region were genotyped in a family-based association study in a sample including 286 offspring with PTB from Morocco (Grant et al. 2013). Stepwise replication and validation in independent population samples from Morocco and Madagascar found association between PTB and a cluster of SNPs with high linkage disequilibrium overlapping the 3′ region of the *TOX* gene. Interestingly, the protein encoded by this gene plays a role in the development of T cells, including CD4+ T cells (Aliahmad et al. 2012). An important finding in this study was that *TOX* association with PTB was driven by early-onset PTB cases (< 25 years old) (Grant et al. 2013). The detection of an age-dependent association highlights the importance of considering age-of-onset in association studies of PTB.

**Table 1:** Summary of loci linked to PTB by genome-wide linkage studies

Reference^a^	Population sample	Locus^b^	Study statistical metrics / Notes^c,d^
Bellamy et al. (2000)	136 South African and Gambian families (83 families in the discovery phase), including 173 sibpairs	15q	LOD score = 2.00, *p* = 0.001
		Xq	LOD score = 1.77, *p* = 0.002
Miller et al. (2004)	38 Brazilian families (16 families in the discovery phase), including 105 affected offspring	10q26.13	LOD score = 1.31, *p* = 0.007
		11q12.3	LOD score = 1.85, *p* = 0.002
		20p12.1	LOD score = 1.78, *p* = 0.002
Baghdadi et al. (2006)	96 Moroccan families (48 families in the discovery phase), including 227 affected siblings	8q12–q13	MLB LOD score = 3.49, *p* = 3 × 10^−5^
Cooke et al. (2008)	105 Malawian and South African families, including 155 affected sibpairs (71 sibpairs from South Africa in the discovery phase)	6p21–q23	LOD score = 1.9, *p* = 0.002
		20q13.31–33	LOD score = 3.1, *p* = 10^−4^
Stein et al. (2008)	193 Ugandan families (95 families in the discovery phase), including 258 full sibling pairs and 175 half sibling pairs	7p22–p21	*p* = 0.0002
		20q13	*p* = 0.002
Mahasirimongkol et al. (2009)	93 Thai families (195 affected individuals)	5q23.2–31.3	LOD score = 2.29, empirical *p* = 0.0005
		17p13.3–13.1	Ordered subset analysis by TB age-of-onset: LOD score = 2.57, permutation *p* = 0.0187
		20p13–12.3	Ordered subset analysis by TB age-of-onset: LOD score = 3.33, permutation *p* = 0.0183

*LOD* logarithm of the odds; *MLB* maximum likelihood binomial

^a^Follow-up association analysis of candidate genes located in the regions linked to the disease are not included in this table

^b^For studies in which the population sample consists of an extension of a previous published GWLS, only the new findings from the extended population analysis are shown

^c^Peak/maximum LOD score is shown for each locus when available

^d^In multi-stage studies, results shown are from the combined analysis with all families when available

A GWLS performed in families from Malawi and South Africa found chromosome regions 6p21–q23 and 20q13.31–33 as PTB susceptibility loci (Table 1) (Cooke et al. 2008). In the same study, 40 SNPs in the 20q13.31–33 region were tested for association in an independent population from West Africa and found variants of the *MC3R* and *CTSZ* genes associated with PTB. Subsequently, variants in both genes were found associated with PTB in an independent case-control population from South Africa (Adams et al. 2011), while only the *MC3R* gene was found associated with disease in an Iranian sample (Hashemi et al. 2013). The linkage hit on chromosome 20q13 was replicated and an additional peak on chromosome 7p22–p21 with suggestive evidence of linkage with PTB was detected in a GWLS of families from Uganda (Stein et al. 2008). A SNP-based GWLS was conducted in 93 multiplex Thai families, including 195 individuals affected with TB (Mahasirimongkol et al. 2009). Chromosome region 5q23.2–31.3 showed evidence of suggestive linkage with TB and two candidate loci on chromosome regions 17p13.3–13.1 and 20p13–12.3 showed evidence of linkage with earlier onset of TB (Table 1). Recently, a 1Mbp region underlying the linkage peak on 20p13–12.3 was sequenced in 13 TB cases from the GWLS Thai families to select functional candidate variants to be tested in association studies in case-control samples from Thailand (Nakauchi et al. 2016). Variants in the *ITPA* gene were identified as risk factor for TB in patients with age-of-onset younger than 45 years of age.

### Genome-wide association studies

The first genome-wide association study (GWAS) for PTB was conducted in 2237 cases and 3122 controls from Ghana and The Gambia followed by stepwise replication of SNPs with suggestive significance in two populations from Ghana and one from Malawi (Table 2) (Thye et al. 2010). A single SNP on chromosome region 18q11.2 within a non-coding chromosomal segment was identified as susceptibility locus for TB. When the genotypes of additional variants were imputed and included in the analysis of the genome-wide Ghanaian data, a TB-protective locus was found on chromosome 11p13 downstream of the *WT1* gene (Table 2) (Thye et al. 2012). The association signal in 11p13 was validated in Gambian and Russian population samples (Thye et al. 2012), as well as in a South African (Chimusa et al. 2014) and a Moroccan sample (Grant et al. 2016). In contrast, only a trend toward association was found in Indonesian patients (Thye et al. 2012). On the other hand, validation of the GWAS findings for 18q11.2 in different populations proved more difficult. This locus was found associated with TB in a Han Chinese cohort (Wang et al. 2013), but in the opposite direction of association than the first TB GWAS (Thye et al. 2010). No association was found in three additional independent Chinese population samples (Dai et al. 2011; Ji et al. 2013; Miao et al. 2016) or in a South African sample (Chimusa et al. 2014). In a Taiwanese sample, association of rs4331426 was found only in Taiwanese women (Lee et al. 2016). When meta-analysis was performed including the aforementioned Chinese and Taiwanese populations with a total of 3118 TB patients and 3226 controls, rs4331426 SNP in 18q11.2 was found not to be associated with TB (Miao et al. 2016). Conflicting results for this locus were also found between a family-based and a case-control sample of a PTB GWAS employing a Moroccan sample (Grant et al. 2016).

**Table 2:** Summary of GWAS hits in PTB

References^a^	Population sample	Locus, candidate gene(s)^b^	Study statistical metrics/notes^c,d^
Thye et al. (2010)	Ghanaian GWAS: 921 cases and 1740 controls. Gambian GWAS: 1316 cases and 1382 controls. Rep I: 1076 cases and 1611 controls (Ghanaian). Rep II: 150 cases and 2214 controls (Ghanaian), 236 cases 779 controls (Malawian)	18q11.2	rs4331426, OR = 1.19 (1.12–1.26), *p* = 6.8 × 10^−9^
Thye et al. (2012)	Ghanaian GWAS: 1329 cases and 1847 controls. Rep I: 817 cases and 3805 controls (Ghanaian). Rep II: 1207 cases and 1349 controls (Gambian), 1025 cases and 983 controls (Indonesian), 4441 cases and 5874 controls (Russian)	11p13, *WT1*	rs2057178, OR = 0.77 (0.71–0.84), *p* = 2.63 × 10^−9^ (GWAS + Rep 1). Meta-analysis *p* = 2.57 × 10^−11^
Png et al. 2012	Indonesian GWAS: 108 cases and 115 controls. Rep I: 600 cases and 540 controls (Indonesian). Rep II: 1837 cases and 1779 controls (Russian)	*TMEFF2*	rs10497744, OR = 0.87 (0.80–0.95), *p* = 0.0014
		*EBF1*	rs10515787, OR = 0.79 (0.68–0.91), *p* = 0.001
		*HAUS6*	rs10245298, OR = 1.23 (1.06–1.41), *p* = 0.005
		*PENK*	rs6985962, OR = 1.17 (1.05–1.31), *p* = 0.006
		*TXNDC4*	rs1418267, OR = 1.13 (1.03–1.23), *p* = 0.007
		*CCL17*	rs188872, OR = 0.87 (0.80–0.95), *p* = 0.002
		*DYNLRB2*	rs4461087, OR = 1.18 (1.07–1.30), *p* = 0.001
		*JAG1*	rs2273061, OR = 1.16 (1.07–1.26), *p* = 0.0004
Mahasirimongkol et al. (2012)	Thai GWAS: 433 cases and 295 controls. Japanese GWAS: 188 cases and 934 controls. Rep: 369 cases and 439 controls (Thai), 112 cases and 1089 controls (Japanese)	20q12, *HSPEP1* and *MAFB*	TB cases with age-at-onset < 45 years: rs6071980, OR = 1.73 (1.42–2.11), *p* = 6.69 × 10^−8^
Chimusa et al. (2014)	South African Colored GWAS: 642 cases and 91 controls	20 *loci*	24 SNPs in 20 *loci* with suggestive *p-*value (≤ 10^−6^), including 2 SNPs in *WT1* [rs2057178, OR = 0.62 (0.50–0.75), *p* = 2.71 × 10^−6^]
Curtis et al. (2015)	Russian GWAS: 5530 cases and 5607 controls. Rep: 1085 cases and 2865 controls (Russian)	8q24, *ASAP1*	rs4733781, OR = 0.84 (0.80–0.88), *p* = 2.6 × 10^−11^
Grant et al. (2016)	Family-based Moroccan GWAS: 252 parents and 306 offspring (239 affected and 67 unaffected). Rep: 317 cases and 657 controls (Morocco)	*AGMO*	TB cases with age-at-onset < 25 years: rs9169432, OR = 2.73 (1.78–4.18), *p* = 2.0 × 10^−6^
		*WNT5A*	rs358793, OR = 0.68 (0.57–0.82), *p* = 4.0 × 10^−5^
		*FOXP1*	rs6786408, OR = 1.47 (1.23–1.79), *p* = 1.9 × 10^−5^
		*PCDH10*	rs17590261, OR = 6.24 (2.38–16.33), *p* = 2.2 × 10^−5^
Sveinbjornsson et al. (2016)	Icelanders GWAS: 3686 PTB cases, 8162 clinical TB cases, 14,723 *Mtb*-infected cases and > 277,643 controls. Rep: 5530 PTB cases and 5607 controls (Russian), 438 PTB cases and 1009 controls (Croatian)	6p21, *HLA*-*DQA1* and *HLA*-*DRB1*	rs557011, OR = 1.18 (1.13–1.23), *p* = 2.0 × 10^−15^; rs9271378, OR = 0.85 (0.82–0.89), *p* = 3.2 × 10^−15^; rs9272785 (*HLA*-*DQA1 p*.Ala210Thr), OR = 1.18 (1.12–1.25), *p* = 1.9 × 10^−9^
Sobota et al. (2016)	Ugandans and Tanzanians GWAS in HIV + individuals: 267 TB cases and 314 controls	5q33.3, *IL12B*	rs4921437, OR = 0.37 (0.27–0.53), *p* = 2.11 × 10^−8^
Omae et al. (2017)	Thai GWAS data set: 263 cases and 282 controls. Rep: 423 cases and 489 (Thai)	1p13, *CD53* and *LRIF1*	TB cases with age-at-onset > 45 years and non-Beijing lineages of Mtb: rs1418425, OR = 1.74 (1.43–2.12), *p* = 2.54 × 10^−8^

*GWAS* genome-wide association study; *HIV* human immunodeficiency virus; *Mtb Mycobacterium tuberculosis; OR* Odds ratio; *PTB* Pulmonary tuberculosis; *Rep* replication; *TB* tuberculosis

^a^Follow-up association analysis in independent studies are not included in this table

^b^For studies in which the population sample consists of an extension of a previous published GWAS, only the new findings from the extended population analysis are shown

^c^Results for the SNP with the strongest association, showing OR (95% confidence interval) and *p* value

^d^Results from meta-analysis or combined analysis with all population samples tested in the study are shown when available

In a GWAS from Indonesia, nearly 95,000 SNPs were tested for association in 108 TB cases and 115 controls (Png et al. 2012). Stepwise replication was conducted for SNPs with *p* < 0.05 in independent Indonesian and Russian samples. Suggestive evidence of association was found between PTB and SNPs in 8 loci (Table 2), including several that are located within genes of the pro-inflammatory Th1 response IFN-γ pathway. A GWAS conducted in Thai and Japanese populations did not identify any significant genetic risk factors for TB; however, when TB cases were stratified by age-of-onset, an association was found between variants of the *HSPEP1* and *MAFB* genes and TB cases with age-of-onset less than 45 years (Mahasirimongkol et al. 2012). Moreover, to consider the heterogeneity of the pathogen genome, the Thai GWAS data were stratified by *M. tuberculosis* lineage [Beijing (39%) vs. non-Beijing lineages (61%)] (Omae et al. 2017). This approach revealed the association of a SNP on chromosome region 1p13 and late-onset TB caused by bacilli belonging to non-Beijing lineages (Table 2). This SNP is located in an intergenic region between the *CD53* and *LRIF1* genes. Interestingly, *CD53* expression was higher in patients with active TB disease when compared to healthy controls or LTBI cases (Omae et al. 2017).

In a Russian sample, a GWAS for PTB was conducted in which 7.6 million variants—genotyped and imputed—were analyzed for association with PTB in 5530 patients and 5607 controls (Curtis et al. 2015). A total of 11 SNPs located in intronic regions of the *ASAP1* gene showed evidence for genome-wide significance of association with PTB (Table 2). These findings were successfully replicated in a second case-control Russian sample (Curtis et al. 2015). Interestingly, *ASAP1* expression was reduced in dendritic cells (DCs) after stimulation with *M. bovis* BCG and *M. tuberculosis*, and the level of reduction correlated with the genotype of an associated SNP. Moreover, ASAP1-depleted DCs showed slower velocity of migration than control cells in in vitro assays, which might play a role in TB pathogenesis by delaying the adaptive immune response (Curtis et al. 2015). However, SNPs in the *ASAP1* gene were not associated with PTB in Western Chinese and Tibetan population samples (Hu et al. 2016), a family-based GWAS in Morocco (Grant et al. 2016) or a case-control GWAS in Iceland (Sveinbjornsson et al. 2016). In the GWAS conducted in North Africa, 558 individuals in a family-based discovery Moroccan sample were recruited for a GWAS of PTB and suggestive findings were tested for association in an independent case-control Moroccan population sample (Table 2) (Grant et al. 2016). Two intronic SNPs—in the *AGMO* and *FOXP1* genes—and two intergenic SNPs—near *WNT5A* and *PCDH10*, respectively—showed suggestive evidence for association with PTB. The *AGMO* association was found only in the < 25 years old subset. Interestingly, *AGMO* and *FOXP1* are involved in the function of macrophages, cells that play an important role in TB pathogenesis (Grant et al. 2016). The Icelandic GWAS which employed both LTBI and clinical disease as outcomes included 3686 PTB patients, 14,724 M. *tuberculosis*-infected cases (made up of LTBI and clinical TB cases), and nearly 300,000 controls (Sveinbjornsson et al. 2016). Three SNPs in the human leukocyte antigen (HLA) class II region were associated with *M. tuberculosis* infection and/or PTB with modest effect on phenotype expression (Table 2). These findings were validated in Russian and Croatian PTB case-control samples (Sveinbjornsson et al. 2016). Two of those SNPs are located between *HLA*-*DQA1* and *HLA*-*DRB1* while the third SNP is a missense variant (p.Ala210Thr) in the *HLA*-*DQA1* gene that represents the *HLA*-DQA1*03 super allele.

In a recent GWAS, an interesting approach was used which led to the identification of a TB-protective locus with strong effect (Sobota et al. 2016). This study enrolled only HIV-positive individuals from prospective TB cohorts in Uganda and Tanzania, where TB is hyperendemic. After 8-years follow-up, 267 HIV-infected patients developed clinical TB while 314 did not, and these groups were included in the GWAS as cases and controls, respectively. Due to immunosuppression, HIV-positive patients exposed to *M. tuberculosis* are at higher risk to progress to active TB. Hence, identification of HIV-positive individuals in TB hyper-endemic regions that do not develop TB disease provides a strong TB resistance phenotype. Following this hypothesis, the GWAS in Uganda and Tanzania identified a SNP located in an intron of the *UBLCP1* gene and 51 kb downstream from *IL12B* as protective for TB disease in highly susceptible individuals (Table 2). This SNP aligns to a known H3K27Ac histone mark which suggests it is involved in gene expression regulation (Sobota et al. 2016).

So far, a common theme underlying the search for genetic factors controlling susceptibility to TB, no matter the technical platform or biological feature investigated, is the difficulty of replication. Even within a self-contained phenotype as PTB, TB has itself revealed as a heterogeneous disease for which genetic susceptibility factors seem to vary depending on population sample, age-at-diagnosis, and *M. tuberculosis* lineages. Hence a first task is to define clearer TB phenotypes (e.g., TST/IGRA double positives, presence of granulomas, or extent of lung involvement), create study designs with tighter age groups and when possible, take into account the causative *M. tuberculosis* strain. On the genetics side, replication steps should not focus only on associated SNPs since signals that may be lost due to population specific LD pattern. Rather, fine mapping of implicated genes may yield more consistent replications.

### Genome-wide RNA expression analysis

Hypothesis-free transcriptome approaches have rapidly increased the knowledge of molecular events underlying a myriad of phenotypes. In TB, transcriptomics has been used to unravel mechanisms of pathogenesis for both *M. tuberculosis* infection and clinical TB disease. For instance, the study of genome-wide mRNA expression levels before and after infection with *M. tuberculosis* of monocyte-derived dendritic cells (DCs) from healthy individuals by microarray identified 3040 differentially expressed genes (Barreiro et al. 2012). Genes involved in immune responses were among those with the most significant changes in expression levels. Further analysis identified more than 700 genes with a cis-eQTL within 200 kb of their transcriptional start sites, including 96 and 102 genes with cis-eQTL only in infected and non-infected DCs, respectively (so-called response cis-eQTL). When crossing these results with the first TB GWAS (Thye et al. 2010), SNPs with a nominal GWAS *p* value of .05 were enriched among response cis-eQTL, but not among cis-eQTL (Barreiro et al. 2012).

Given the relative ease of sampling, transcriptomic biomarkers obtained from whole blood are attractive targets to derive minimal RNA signatures capable of discriminating resistance to infection or progression from LTBI to active PTB, and several studies tackled these goals aiming for potential applications in clinical settings (Berry et al. 2010; Maertzdorf et al. 2011; Kaforou et al. 2013; Anderson et al. 2014). With focus on progression from LTBI to PTB, a large prospective study screened and followed the clinical evolution of 6363 adolescents infected with *M. tuberculosis* for 6 months to 2 years (Zak et al. 2016). A total of 46 subjects progressed to active PTB and their blood transcriptomes were compared with the transcriptomes of 107 *M. tuberculosis*-infected subjects who remained free of TB. The samples were split in a training and test set and a 16 gene progression signature was identified. The signature predicted progression to active TB disease in a time-frame of up to 18 months prior to clinical disease. The predictive value of the signature increased for subjects that were closer to active disease. These findings were validated in three independent populations. Interestingly, the RNA signature was able to distinguish non-PTB diseases from active PTB (Zak et al. 2016). In a follow-up study, longitudinal transcriptome changes for type I/II interferon response genes, Th17-associated genes, and RNA transcript modules that correlated with different whole blood cellular sub-population were shown to be sequentially modulated over two years in TB progressors (Scriba et al. 2017).

A striking effect of *M. tuberculosis* infection has also been described for whole blood mRNA and miRNA over a 96 h time course in samples from non BCG-vaccinated healthy individuals (von Both et al. 2018). When considering differentially expressed (DE) genes with an absolute log fold change > 1, 75% were down-regulated in response to *M. tuberculosis*. By investigating maximum fold changes in relation to time points for all DE genes, the maximum suppression was achieved within 48 h for genes involved in innate immunity while genes that were down-regulated up to 96 h were those related to adaptive immunity. Independent experiments identified 97 DE miRNAs whose mRNA targets were over-represented among both down-regulated and all DE genes (von Both et al. 2018). Non-coding genes have previously been shown to be differentially expressed following *M. tuberculosis* infection (Siddle et al. 2014), and they have been suggested as putative markers of PTB (Chen et al. 2017) and as modulators of genes involved in autophagy (Etna et al. 2018).

Despite the promising results, study-specific signatures derived from microarray experiments have little or no overlap regarding the genes implicated (Blankley et al. 2016a). To overcome possible sample size limitations from microarray datasets and test new hypotheses, meta-analyses have been performed and identified novel and validated DE genes from individual studies (Blankley et al. 2016a; Wang et al. 2018). However, the number of DE genes is drastically reduced if consistency is required across all datasets. Although no gene signatures were reported, genes detected by meta-analyses implicated host response pathways, such as the type I interferon signaling cascade, that were consistently disturbed in TB. Evidence accumulated from blood transcriptome analyses across the TB pathogenesis spectrum (from infection to active disease) so far suggests that (i) RNA makers reflect various stages of TB progression rather than providing susceptibility makers; and (ii) that TB disease displays significant phenotypic heterogeneity (Scriba et al. 2017; Blankley et al. 2016b). In contrast to PTB, derivation of RNA expression profiles that discern LTBI from non-infected individuals remained a challenge (Wang et al. 2018). Nevertheless, recent work investigated the transcriptomic differences between *M. tuberculosis* infection-resistant and LTBI subjects and implicated histone deacetylase pathways as putative regulators of innate immunity to mycobacteria (Seshadri et al. 2017). The transcriptomic assays mentioned above contributed to the knowledge of host response to *M. tuberculosis* in tissues or cell-types that come in contact with the pathogen/antigens in a series of events that may define infection success. An aspect that deserves more attention is the characterization of cell types that act as first line defense. Alveolar macrophages likely serve this function and studying their response to *M. tuberculosis* in diverse ethnic cohorts may provide further insights (Maertzdorf et al. 2018).

## Leprosy

The etiological agent of leprosy, a chronic infectious disease, is the slow growing *Mycobacterium leprae*, which primarily affects the skin and peripheral nerves. Over the last 5 years the global leprosy incidence reported by the World Health Organization (WHO) was remarkably constant at approximately 210,000 patients (WHO 2017b). However, it is likely that these numbers are a severe underestimate of the true incidence (Smith et al. 2015). Leprosy presents along a spectrum of clinical phenotypes that are being classified employing different criteria (Gaschignard et al. 2016). Based on the WHO leprosy classification system, leprosy patients are classified as paucibacillary (PB) when presenting up to five lesions, or multibacillary (MB) when presenting more than five lesions. An important aspect of leprosy is that during or after treatment, some patients can manifest acute episodes of dysregulated inflammation known as leprosy reaction. As result of this condition, patients may experience pain and develop severe nerve damage and permanent sequelae. The most common form of reactional state is type 1 reaction (T1R), which can affect 30–50% of leprosy patients depending on the epidemiologic setting (Fava et al. 2012). There is accumulating evidence that human genetics plays an important role in leprosy susceptibility, with different sets of genes modifying host predisposition to leprosy per se, its clinical forms and reactional states (Sauer et al. 2015; Fava and Schurr 2016).

### Genome-wide linkage studies

The first GWLS in leprosy detected a linkage peak on chromosome region 10p13 in Indian families with PB leprosy patients (Table 3) (Siddiqui et al. 2001). A subsequent genomic scan in a sample of Vietnamese families replicated linkage to the same region with PB leprosy (Mira et al. 2003). Based on these findings, the *MRC1* gene—located in the linked chromosomal interval—was selected as positional and functional candidate for association study since the protein encoded by this gene is a receptor for uptake of mycobacteria. Unexpectedly, in Vietnamese and Brazilian leprosy patients, *MRC1* variants were associated with leprosy per se and MB leprosy, but not PB leprosy (Alter et al. 2010). In 2014, a gene-centered high-density association scan of the chromosomal 10p13 interval was conducted in two independent family-based population samples from Vietnam (Grant et al. 2014). Two independent association signals in the *CUBN* and *NEBL* genes were detected and, again, the association of both genes was with the MB clinical form. Additionally, the *GATA3* gene, located 6.5 Mb from the linkage peak on chromosome 10p13, was tested for association with leprosy and its clinical forms in two Brazilian population samples and a single SNP was associated with leprosy per se in both samples (Medeiros et al. 2016). Combined, these association studies identified one neighboring and three 10p13 genes involved in the control of leprosy susceptibility and the MB clinical form. Why genetic studies failed to detect an association with PB leprosy is perplexing and not known. It is possible that a collection of rare variants within this region impacts on the risk of PB leprosy but presently there are no deep sequencing data available to test this hypothesis (Orlova et al. 2011; Grant et al. 2014).

**Table 3:** Summary of loci linked to leprosy by genome-wide linkage studies

References^a^	Population sample	Locus^b^	Study statistical metrics/notes^c,d^
Siddiqui et al. (2001)	224 Indian families (93 families in the discovery phase), including 245 sibpairs	10p13	Multipoint LOD score = 4.09, *p* = 0.000007
Tosh et al. (2002)	233 Indian families (175 families from Tamil Nadu; 93 families in the discovery phase), including 256 sibpairs	20p12	Single-point LOD score = 3.39, *p* = 0.00003, multipoint LOD score = 1.29 (Single-point LOD score = 3.48 and multipoint LOD score = 3.16 in families from Tamil Nadu)
Mira et al. (2003)	86 Vietnamese families, including 205 affected siblings. Leprosy subtypes: 10 MB families and 17 PB families	6p21	Multipoint MLB LOD score = 2.62, *p* = 2.5 × 10^−4^
		6q25-q27	Multipoint MLB LOD score = 4.31, *p* = 5 × 10^−6^
		10p13	PB: multipoint MLB LOD score = 1.74, *p* < 0.003; MB: multipoint MLB LOD score = 0.01. Heterogeneity *p* = 0.028
Miller et al. (2004)	71 Brazilian families (21 families in the discovery phase), including 191 affected offspring. Leprosy subtypes: 44 LL families and 39 TT families	6p21.32	LOD score = 3.23, *p* = 5.8 × 10^−5^
		17q22	LOD score = 2.38, *p* = 0.0005
		20p13	LOD score (D20S889) = 1.51, *p* = 0.004. LL: LOD score (D20S889) = 1.36, *p* = 0.006. TT: LOD score (D20S835) = 0.74, *p* = 0.033
Wallace et al. (2004)	185 extended families from Malawi (83 nuclear families in the discovery phase)	21q22	LOD score = 1.4, *p* ≈ 0.001
Yang et al. (2012)	23 Chinese families, including 82 affected individuals	2p14	HLOD = 3.51 (REC), LOD score = 1.48, *p* = 0.005
		4q22	HLOD = 2.92 (DOM), LOD score = 1.21, *p* = 0.009
		8q24	HLOD = 2.74 (REC), LOD score = 1.22, *p* = 0.009
		16q24	HLOD = 1.93 (DOM), LOD score = 1.37, *p* = 0.006
		6q24-q26	LOD score = 1.54, *p* = 0.004

*DOM* dominant model; *HLOD* heterogeneity logarithm of odds; *LOD* logarithm of the odds; *LL* lepromatous leprosy; *MB* multibacillary leprosy; *MLB* maximum likelihood binomial; *PB* paucibacillary leprosy; *REC* Recessive model; *TT* tuberculoid leprosy

^a^Follow-up association analysis of candidate genes located in the regions linked to the disease are not included in this table

^b^For studies in which the population sample consists of an extension of a previous published GWLS, only the new findings from the extended population analysis are shown

^c^Peak/maximum LOD score is shown for each locus when available

^d^In multi-stage studies, results shown are from the combined analysis with all families when available

In addition to the linkage peak in the 10p13 region, chromosome region 6q25–q27 was linked to leprosy per se susceptibility in the same Vietnamese families (Table 3) (Mira et al. 2003). A fine mapping association study of 43 genes located in the 6q25–q27 region identified several SNPs located within the promoter region shared by two genes—*PRKN* (formerly *PARK2*, a well-known early onset Parkinson disease gene) and *PACRG—*associated with leprosy susceptibility in Vietnamese families (Mira et al. 2004). These results were validated in a Brazilian sample (Mira et al. 2004), but not in an Indian (Malhotra et al. 2006) and a Chinese sample (Li et al. 2012). Later, two studies performed high-density association mapping of the *PRKN*/*PACRG* regulatory region in independent population samples to analyze the impact of ethnic background on the association (Alter et al. 2013; Chopra et al. 2013). Both studies confirmed association and revealed that differences in linkage disequilibrium (LD) patterns across different ethnicities may explain the heterogeneity of association in previous studies between this locus and leprosy per se. Moreover, Alter et al. also demonstrated that the *PRKN*/*PACRG* association with the disease is dependent on the age-at-diagnosis: a more pronounced genetic effect is found in early-onset patients (Alter et al. 2013). Interestingly, the role of Parkin—the protein encoded by the *PRKN* gene—in host response against intracellular parasites has been demonstrated by functional assays. In different animal models, it was demonstrated that Parkin plays a role in the pathway that leads to degradation of intracellular pathogens—such as mycobacteria, salmonella and listeria—across distantly related species (Manzanillo et al. 2013). In addition, abrogation of *PRKN* in macrophages and Schwann cells affected their ability to produce IL-6 and CCL2—two key pro-inflammatory cytokines—in response to mycobacteria and lipopolysaccharides (de Léséleuc et al. 2013). Besides the *PRKN*/*PACRG* association, a recent study found a new gene located at chromosome 6q25-27—the *SOD2* gene—as a risk factor for leprosy susceptibility in two independent Brazilian population samples (Ramos et al. 2016). Indeed, *SOD2* expression was downregulated in the human acute monocytic leukemia cell lineage (THP-1) cells after stimulation with live *M. leprae* (Guerreiro et al. 2013).

The GWLS conducted in the Vietnamese families detected an additional leprosy linkage signal at the HLA complex on chromosomal region 6p21 (Table 3) (Mira et al. 2003). This region had previously been linked to leprosy susceptibility in a Brazilian sample (Miller et al. 2004). In fact, several studies have reported the involvement of HLA alleles and haplotypes as important genetic factors controlling susceptibility to leprosy (Geluk and Ottenhoff 2006; Jarduli et al. 2013). To further explore the region underlying the linkage peak at 6p21, a stepwise association study was conducted to scan a 10.4 Mb region that encompasses 224 annotated genes within and centromeric to the HLA class II and class III regions (Alcaïs et al. 2007). A functional SNP was identified in the *LTA* promoter region as a risk factor of leprosy in ethnically distinct populations. Interestingly, the *LTA* genetic effect on leprosy risk was age dependent, since evidence for association became stronger with early age-at-diagnosis. To identify additional genetic risk factors for leprosy in the 6p21 chromosomal region, a high-density SNP association scan was conducted on a 1.9 Mb chromosomal underlying the HLA complex (Alter et al. 2011). Employing samples from Vietnam and North India, SNPs in the HLA class I region were found associated with leprosy per se and strongly implicated the HLA-C*15:05 allele in leprosy susceptibility. In addition to the above-mentioned genes in the HLA region, cumulative evidence indicated that the class III gene TNF was also involved in the immune response against leprosy. Specifically, a promoter variant located at position − 308 of TNF has been extensively studied in leprosy, with inconsistent results (Cardoso et al. 2011b). Interestingly, a large association study conducted in four Brazilian population samples, followed by a meta-analysis including additional genotypes from published data, reinforced the TNF − 308 protective effect in leprosy and suggested that the association is restricted to the Brazilian population (Cardoso et al. 2011a).

Complementary to these findings, several GWLS have reported other chromosomal regions involved in the control of leprosy phenotypes (Table 3) (Fava and Schurr 2016). Further analysis of the first leprosy GWL data with an extended Indian population found an additional linkage peak on chromosomal region 20p12 (Tosh et al. 2002). In Brazilian families with leprosy patients, suggestive evidence of linkage was found with the 17q22 and 20p13 chromosome regions (Miller et al. 2004). The latter linkage peak was located 3.5 Mb distal to the linkage peak reported in the Indian sample. Moreover, chromosomal region 21q22 was linked to leprosy polarization in families from Malawi (Wallace et al. 2004). Finally, 2p14 was significantly linked to leprosy per se, while regions 4q22, 8q24 and 16q24 showed suggestive evidence for linkage in Chinese families (Yang et al. 2012). Leprosy susceptibility genes underlying these linkage peaks are yet to be identified.

### Genome-wide association studies

The first leprosy GWAS was conducted in 2009 in four independent Chinese case-control samples (Zhang et al. 2009). Among the nearly 500,000 SNPs tested in the first population, 93 SNPs were associated with leprosy and reanalyzed in the three remaining case-control samples. In total, 15 SNPs located in five loci—*HLA*-*DR*–*DQ, RIPK2, TNFSF15, NOD2* and *CCDC122-LACC1*—were significantly associated with leprosy (*p* < 1.00 × 10^−10^; Table 4). Moreover, a trend toward association was found for one SNP near the *LRRK2* gene (Table 4). Of note, most of these genes are implicated in Crohn’s disease (Schurr and Gros 2009; Zhang et al. 2009; Grant et al. 2012). After the leprosy GWAS results, several association studies attempted to replicate these genes in independent populations. The *NOD2* association with leprosy was validated in a Nepalese population, where it was also found associated with leprosy reaction (Berrington et al. 2010). *HLA*-*DR*–*DQ* and *CCDC122*–*LACC1 loci* were associated with disease in population samples from India and West Africa (Wong et al. 2010b, a). Moreover, *RIPK2* was validated in an Indian population (Marcinek et al. 2013), while *LRRK2* was found to be associated with leprosy and the PB clinical form in Chinese and Indian population samples (Marcinek et al. 2013; Wang et al. 2015).

**Table 4:** Summary of GWAS hits in leprosy phenotypes

References^a^	Population sample	Locus, candidate gene(s)^b^	Study statistical metrics/notes^c,d^
Zhang et al. (2009)	Chinese GWAS: 706 cases and 1225 controls. Rep: 2164 cases and 4373 controls (Chinese), 304 cases and 709 controls (Chinese), 786 cases and 873 controls (Chinese)	*HLA*-*DR*–*DQ*	rs602875, OR = 0.67 (0.62–0.72), *p* = 5.35 × 10^−27^
		*RIPK2*	rs42490, OR = 0.76 (0.71–0.81), *p* = 1.38 × 10^−16^
		*TNFSF15*	rs6478108, OR = 1.37 (1.28–1.46), *p* = 3.39 × 10^−21^
		*LRRK2*	rs1873613, OR = 0.86 (0.80–0.92), *p* = 5.10 × 10^−5^
		*CCDC122*	rs3088362, OR = 1.52 (1.41–1.63), *p* = 1.36 × 10^−31^
		*LACC1* (*C13orf31*)	rs3764147, OR = 1.68 (1.57–1.80), *p* = 3.72 × 10^−54^
		*NOD2*	rs9302752, OR = 1.59 (1.49–1.71), *p* = 3.77 × 10^−40^
Zhang et al. (2011)	Chinese GWAS: 706 cases and 5581 controls. Rep: 2307 cases and 4585 controls (Chinese), 273 cases and 214 controls (Chinese), 721 cases and 500 controls (Chinese)	1p31.3, *IL23R*	rs3762318, OR = 0.69 (0.62–0.77), *p* = 3.27 × 10^−11^
		6q24.3, *RAB32*	rs2275606, OR = 1.30 (1.21–1.39), *p* = 3.94 × 10^−14^
Liu et al. (2015)	Chinese GWAS (I): 706 cases and 5587 controls. Chinese GWAS (II): 842 cases and 925 controls. Rep I: 2761 cases and 3038 controls (Chinese). Rep II: 4004 cases and 6467 controls (Chinese)	1q32.3, *BATF3*	rs2221593, OR = 1.15, *p* = 3.09 × 10^−8^
		5p14.3, *CDH18*	rs73058713, OR = 1.19, *p* = 9.54 × 10^−9^
		9q32, *DEC1*	rs10817758, OR = 1.13, *p* = 1.15 × 10^−8^
		10q21.3, *EGR2*	rs58600253, OR = 1.22, *p* = 3.02 × 10^−12^
		11q13.1, *CCDC88B*	rs663743, OR = 1.24, *p* = 8.84 × 10^−14^
		*16p13.13, CIITA* and *SOCS1*	rs77061563, OR = 0.84, *p* = 6.23 × 10^−15^
Wang et al. (2016)	Chinese GWAS (I): 706 cases and 1223 controls. Chinese GWAS (II): 840 cases and 924 controls. Chinese GWAS (III): 1197 cases and 1426 controls. Rep I: 1516 cases and 1512 controls. Rep II: 3897 cases and 10525 controls (Chinese)	3p25.2, *SYN2*	rs6807915, OR = 0.89, *p* = 1.94 × 10^−8^
		7p14.3, *BBS9*	rs4720118, OR = 1.16, *p* = 3.85 × 10^−10^
		8p23.1, *CTSB*	rs55894533, OR = 1.15, *p* = 5.07 × 10^−11^
		8q24.11, *MED30*	rs10100465, OR = 0.85, *p* = 2.85 × 10^−11^
Liu et al. (2017)	Chinese GWAS of protein-coding variants: 1648 cases and 2318 controls. Rep I: 3169 patients and 9814 controls (Chinese). Rep II: 2231 patients and 2266 controls (Chinese)	*IL23R*	rs76418789 (G149R), OR = 1.36 (1.24–1.49), *p* = 1.03 × 10^−10^
		*FLG*	rs146466242 (K4022*), OR = 1.45 (1.31–1.61), p* = 3.39 × 10^−12^
		*NCKIPSD*	rs145562243 (R176Q), OR = 4.35 (2.58–7.35), *p* = 1.44 × 10^−8^ (GWAS + Rep I). Meta-analysis *p* = 1.71 × 10^−9^
		*CARD9*	rs149308743 (R494H), OR = 4.75 (2.87–7.86), *p* = 4.99 × 10^−10^ (GWAS + Rep I). Meta-analysis *p* = 2.09 × 10^−8^
		*SLC29A3*	rs780668 (S158F), OR = 1.14 (1.09–1.19), *p* = 2.17 × 10^−9^
		*IL27*	rs181206 (L119P), OR = 0.83 (0.78–0.89), *p* = 1.08 × 10^−7^
		*TYK2*	rs55882956 (R703W), OR = 1.30 (1.17–1.45), *p* = 1.04 × 10^−6^
Fava et al. (2017a)	Vietnamese GWAS in T1R: 221 families with 229 leprosy T1R-affected offspring and 209 families with 229 leprosy T1R-free offspring. Rep: 253 T1R cases and 563 T1R-free controls (Vietnamese). Validation: 471 T1R cases and 446 T1R-free controls (Brazilians)	10p21.2, *LOC105378318*/*ENSG00000235140*	T1R: rs1875147, OR = 1.54 (1.32–1.80), *p* = 4.5 × 10^−8^

*OR* odds ratio; *Rep* replication; *T1R* type 1 reaction

^a^Follow-up association analysis in independent studies are not included in this table

^b^For studies in which the population sample consists of an extension of a previous published GWAS, only the new findings from the extended population analysis are shown

^C^Results for the SNP with the strongest association, showing OR (95% confidence interval) and *p* value when available

^d^Results from meta-analysis or combined analysis with all population samples tested in the study are shown when available

In a family-based Vietnamese sample, 16 significant GWAS SNPs were tested for association and SNPs tagging *HLA-DR–DQ, RIPK2, NOD2* and *CCDC122–LACC1* were validated as risk factors for leprosy susceptibility (Grant et al. 2012). Interestingly, when these families were stratified by the T1R status of leprosy patients, variants located in two genes that could not be replicated for leprosy per se—*TNFSF15* and *LRRK2*—were found associated with T1R (Fava et al. 2015, 2016). A total of 47 SNPs within *TNFSF15* and the adjacent *TNFSF8* were found associated with T1R in Vietnamese patients. Moreover, 83% of SNPs were associated with *TNFSF8* gene transcript levels in multiple tissues, indicating that, in fact, the association with T1R might be due to this gene and not *TNFSF15* (Fava et al. 2015). One of the SNPs showed evidence of an age-dependent genetic effect since the risk effect for T1R was stronger in younger than 30 years old patients (OR = 1.95, 95% CI = 1.54–2.46, combined *p* = 2.5 × 10^−8^) than in the global population sample (OR = 1.46, 95% CI = 1.23–1.73, combined *p* = 1.5 × 10^−5^) (Fava et al. 2017b). In *LRRK2*, the main SNP capturing the T1R association was a missense variant (M2397T) (Fava et al. 2016). This coding variant was known to impact LRRK2 protein turnover—where the protein with the M2397 allele presents shorter half-life than 2397T—and was previously reported in association with Crohn’s disease with the same risk allele as in T1R (M2397) (Liu et al. 2011; Fava et al. 2016). Subsequent eQTL analysis showed that nine variants belonging to the same SNP bin as M2397 promote an increase in *LRRK2* expression only in non-stimulated cells. This indicated that these eQTL SNPs counterbalanced LRRK2 shorter half-life due to the M2397 variant by increasing mRNA expression levels. However, this compensatory mechanism was abrogated following stimulation with *M. leprae* (Fava et al. 2016). A stepwise association study of leprosy and the non-HLA genes that were significantly associated in the GWAS (*RIPK2, TNFSF15, NOD2* and *CCDC122-LACC1*) was conducted in four independent Brazilian population samples (Sales-Marques et al. 2014). Initially, 36 SNPs were genotyped, capturing the complete information of the five genes, in a family-based population sample from Prata Village—an isolated, leprosy hyper-endemic population located in the Brazilian Amazon. Two SNPs located in *NOD2* and *CCDC122-LACC1* were associated with leprosy and were subsequently replicated in three independent Brazilian case-control population samples (Sales-Marques et al. 2014).

In 2011, an expanded analysis of the first GWAS (Zhang et al. 2009) was performed by combining the GWAS data set with additional control subjects leading to the identification of *IL23R* and *RAB32* as additional leprosy associated genes in the Chinese sample (Table 4) (Zhang et al. 2011). Association of both genes was validated in Vietnamese families (Cobat et al. 2014). In two subsequent studies, the Chinese GWAS data set was further expanded (Table 4) (Liu et al. 2015; Wang et al. 2016). First, six additional loci were associated with leprosy when a second independent Chinese GWAS dataset was added to the previous GWAS dataset, followed by a two-stage replication in Chinese case-control samples (Table 4) (Liu et al. 2015). Finally, a third GWAS dataset was created by including results from a population-specific array in a three-stage GWAS meta-analysis comprising 8156 cases and 15,610 controls of Chinese ancestry (Wang et al. 2016). In addition to confirming all loci identified in the previous GWAS (Zhang et al. 2009, 2011; Liu et al. 2015), four novel loci were associated with leprosy (Table 4) (Wang et al. 2016). Finally, a genome-wide association study of protein coding genes and leprosy susceptibility has been conducted in a Chinese sample (Liu et al. 2017). In this study, 40,491 coding variants with minor allele frequency greater than 0.1% were tested for association in a discovery set composed of 3966 individuals (1648 cases). Then, stepwise replication was conducted in four independent Chinese population samples which led to identification of seven nonsynonymous variants that were associated with leprosy. These findings implicated six new genes in disease susceptibility—*CARD9, FLG, IL27, NCKIPSD, SLC29A3* and *TYK2*—and also confirmed the association of the *IL23R* GWAS gene (Table 4).

Until now, only one GWAS for T1R risk in leprosy patients has been published. It was conducted in a family-based population sample composed of 221 Vietnamese families with 229 offspring with leprosy that were also T1R-affected and 209 families with 229 offspring with leprosy that were T1R-free (Fava et al. 2017a). In this GWAS, 6.3 million variants—genotyped and imputed—were tested for association in both family sets independently, followed by a heterogeneity test to detect associations that are specific to the T1R-affected subset. SNPs located between two recombination hot-spots on chromosome 10p21.2 were found preferentially associated with T1R. Stepwise replication of these findings in two independent case-control populations from Vietnam and Brazil allowed to narrow the association signal to a single eQTL SNP for a lncRNA gene as a risk factor for T1R (OR = 1.54, 95% CI 1.32–1.80, combined *p* = 4.5 × 10^−8^) (Fava et al. 2017a).

GWLS, GWAS, and follow-up studies have contributed substantially to a better understanding of the human genetic factors involved in leprosy susceptibility. Moreover, not only have these studies led to the identification of genes and pathways that play a role in leprosy pathogenesis, but they have also identified covariates that may be critical for the genetic analyses of other complex diseases. For example, age-at-diagnosis, population samples size and phenotypic homogeneity, differences in LD pattern, and the effect of endophenotypes—for leprosy, T1R and leprosy subtypes—all can impact on the detection and interpretation of genetic findings.

### Genome-wide RNA expression analysis

RNA expression analyses have provided valuable data to better understanding leprosy pathogenesis. Comparisons of mRNA and miRNA expression between leprotic lesions and skin biopsies of healthy controls showed a large number of genes differentially expressed, including DE mRNAs and miRNAs exclusively detected in samples with leprosy reactions (Belone et al. 2015; Soares et al. 2017). Moreover, contrasting nerve biopsies of leprosy lesions against non-leprous neuropathy ones highlighted down-regulated cytokines and genes involved in mitochondrial metabolism as mediators of the host-*M. leprae* interplay (Guerreiro et al. 2013).

Recently, the impact of genetic variation on the human response to *M. leprae* was demonstrated by an eQTL study of whole blood cells in the presence and absence of *M. leprae* antigen (Manry et al. 2017). Of particular interest was the identification of genetic regulators of host gene expression levels that depended on the presence of *M. leprae*. Specifically, stimulation of whole-blood from leprosy patients with *M. leprae* sonicate identified 6675 genes differentially expressed (*p* < 4.2 × 10^−6^); 35% being up-regulated. Gene ontology term analysis showed that the majority of over-expressed genes were involved in immune system processes and immune related functions. Next, 1.7 million variants with minor allele frequencies > 10% and located within a 200 kb window of gene transcription start sites were tested for cis-eQTL effects. This led to the identification of 318 genes that were impacted by *cis*-eQTL in either stimulated or non-stimulated samples (FDR of 0.01), while 66 of these genes displayed eQTL only in one condition (response eQTL) directly demonstrating the interaction of host genetic background and pathogen exposure. Interestingly, the same study also showed that both eQTL and response-eQTL are targeted by positive selection (Manry et al. 2017).

In an effort to derive transcriptome biomarkers for those leprosy patients who are at increased risk of developing T1R, the transcriptome response to *M. leprae* sonicate in whole blood from leprosy patient was compared between those patients who developed T1R and those who did not (Orlova et al. 2013). Employing retrospectively enrolled (i.e. cured) patients, compared to T1R-free patients a set of 44 genes was found preferentially upregulated in T1R patients of which 32 could be assigned to three functional groups: pro-inflammatory regulators, arachidonic acid metabolism mediators and regulators of anti- inflammation. Importantly, differentially upregulated genes allowed to derive a gene-set signature for T1R. The T1R signature was validated in a prospective study where blood from newly diagnosed T1R-free leprosy patients was stimulated with *M. leprae* antigen prior to the extraction of RNA. All patients were then followed for three years and the occurrence of T1R was recorded (Orlova et al. 2013). The enrichment of pro- and anti-inflammatory mediators among the T1R signature gene expression set prior to T1R onset suggests an innate defect in the regulation of the inflammatory response to *M. leprae* antigens.

## Buruli ulcer

The third most common human mycobacterial disease is BU, a necrotizing skin disease caused by *Mycobacterium ulcerans*, a toxin-producing environmental mycobacterium. The clinical presentations of BU are non-ulcerative or ulcerative lesions or osteomyelitis, located mainly in the limbs. Up to 22% of BU patients develop permanent functional sequelae, including amputations, which is associated with severe BU (presentation with large lesions, edema, osteomyelitis, or multifocal lesions) (Vincent et al. 2014). Cases of BU have been detected in more than 30 countries in Africa, the Western Pacific and South America. While, estimates for global incidence are presently not available (WHO 2017c), in 2016 only ten countries reported nearly 2000 BU cases to WHO (WHO 2017d). The disease is classified by WHO in three categories in terms of severity: (i) category I if the patient has one small lesion measuring < 5 cm, (ii) category II if the patient presents non-ulcerative or ulcerative plaque, edema or one large lesion with maximum diameter between 5 and 15 cm, and (iii) category III if presenting disseminated or mixed forms, multiple lesions or one extensive lesion measuring > 15 cm diameter (WHO 2012, 2017c). In some BU patients, a condition called paradoxical reaction (PR) can occur following the antibiotic treatment of *M. ulcerans*. PRs are characterized by intense inflammation in lesions and worsening clinical conditions after initial improvement (O’Brien et al. 2013).

### Candidate gene association studies

As in TB and leprosy, following exposure of *M. ulcerans* there is a variability of outcome regarding both disease per se and the clinical manifestations of BU, suggesting that immune and genetic risk factors could play a role in this disease as well (Stienstra et al. 2001; Portaels et al. 2009; Vincent et al. 2014). This conclusion is further supported by family history of BU as risk factor for the disease (Sopoh et al. 2010). Fueled by these observations, association studies were designed to test candidate genes based on their previous implications in TB and/or leprosy and analysis of the implicated pathways. The first association study in BU was published in 2006 employing a Ghanaian case-control population sample (Stienstra et al. 2006). In this study, three *SLC11A1* (formerly *NRAMP1*) variants were tested for association and one SNP was found significantly associated with BU susceptibility (OR = 2.89, *p* = 0.004). Later, a case-control association study of nine SNPs in three autophagy-related genes—*PRKN, NOD2* and *ATG16L1*—was conducted in a population sample from Benin (Capela et al. 2016). The SNP rs1333955 in *PRKN* displayed an odds ratio of 1.43 (*p* = 0.05) risk of BU. The same SNP had previously been identified as main cause of the association of *PRKN* with leprosy and also was shown to be a trans-eQTL for *CCL2* and *IL6* (de Léséleuc et al. 2013; Alter et al. 2013).

Considering severity of disease, two association signals in *NOD2* were found for susceptibility to WHO category 3 when compared to combined category 1 and 2 [rs9302752 OR = 2.23, *p* = 0.02 and rs2066842 (P268S) OR = 12.7, *p* = 0.03]. Finally, *ATG16L1* was found associated with protection from presenting the ulcerative phenotype (OR = 0.35, *p* = 0.02). In 2016, four variants in *SLC11A1* were genotyped in BU patients with clinical diagnosis of paradoxical reactions and BU patients without reaction from Ghana (Barogui et al. 2016). A TGTG indel located in the *SLC11A1* 3′UTR was associated with paradoxical reaction, revealing the homozygous ins/ins genotype as a risk factor to develop PR (OR = 7.19, *p* < 0.001).

Recently, a case-control association study for BU and nine SNPs in six genes was performed in a case-control population sample from Ghana (Bibert et al. 2017). A trend toward association of a previously associated *SLC11A1* SNP and BU predisposition was found (OR = 1.63, *p* = 0.06). In addition, SNPs in two genes were found as new candidates for BU risk factors: *IFNG* rs2069705 (OR = 1.56, *p* = 0.007) and *NOS2* rs9282799 (OR = 1.99, *p* = 0.006). No association was detected between *NOD2, PRKN* and *VDR* SNPs and BU susceptibility. Moreover, a variable nucleotide tandem repeat (VNTR) located in intron 1 of *IFNG*, known to be in LD with rs2069705 was tested. When the two shorter length CA repeats were combined into a single allele, association to BU was found (OR = 0.60, *p* = 0.007). Moreover, since *IFNG* rs2069705 and *NOS2* rs9282799 are located in the promoter regions of these genes, in vitro assays demonstrated that both SNPs have an impact on gene expression. Interestingly, the *IFNG* SNP only impacted on mRNA and protein expression levels following *M. ulcerans* but not *M. marinum* stimulation (Bibert et al. 2017).

Together, these candidate association studies provided initial evidence that host genetic factors play a role in predisposing to BU and its clinical subtypes. Still, replication and validation studies in independent populations are necessary to clarify the involvement of these genes in susceptibility to BU. Regarding candidate-free approaches, a recent study applied GWLS in a consanguineous family from Benin presenting two cases with extreme BU phenotype, which led to the identification of a region on chromosome 8 in linkage with the disease (Vincent et al. 2018). Exome sequencing was carried out on the affected siblings and a 37 kb homozygous deletion was found that overlapped a lincRNA and was located near a beta-defensin cluster. Next, data from an array scan in a cohort of 402 BU cases and 401 controls from Benin was queried for the deletion which was found in two homozygous unrelated BU patients while none of the controls were homozygous for the deletion (Vincent et al. 2018). These findings further support the presence of genetic factors controlling susceptibly to BU and given the few studies in this disease, population-based candidate-free strategies are called for and will likely shed additional light on pathogenic mechanisms of BU.

## Conclusion

In the last decade, human genetics of TB and leprosy susceptibility were extensively studied by genome-wide approaches to complement findings from candidate gene studies in the search of host genetic control of disease susceptibility. Major disease susceptibility loci have been mapped to human chromosome regions by GWLS, and, at least for some examples, this was followed by a search for genes that underlie those loci leading to the identification of new candidate susceptibility genes. Regarding GWAS data and replications of those findings, TB and leprosy studies surprised in different ways. In TB, the number of genetic risk factors and their effect size on susceptibility were smaller than expected and replication in different population proved a difficult task. The reason for that is not clear, but it could be due to the heterogeneity of the phenotype, to host-environment interactions or to the variability of the *M. tuberculosis* genome. In leprosy, GWAS and replication studies provided insights into disease pathogenesis and revealed an unexpected overlap in the genetic control of leprosy and its clinical presentations with common inflammatory disorders such as Crohn’s disease. In addition, genome-wide RNA expression analyses in mycobacterial disease contributed valuable data of the host-pathogen interplay leading to gene signatures that might be useful for disease control. These studies also detected new genes and pathways that might help to better understand the cellular response to infection and clinical disease. Finally, despite the debilitating outcomes of BU and perhaps due to lower disease burden, research of the genetics of BU susceptibility is still in its infancy. Application of large scale genome-wide approaches to BU may contribute to a better understanding of the role of human genetic factors in predisposition to BU and hopefully pave the way for an effective control of this condition.
